# Chromosomal instability induced by increased *BIRC5*/Survivin levels affects tumorigenicity of glioma cells

**DOI:** 10.1186/s12885-017-3932-y

**Published:** 2017-12-28

**Authors:** Marina Conde, Susanne Michen, Ralf Wiedemuth, Barbara Klink, Evelin Schröck, Gabriele Schackert, Achim Temme

**Affiliations:** 1 Department of Neurosurgery, Section Experimental Neurosurgery/Tumor Immunology, University Hospital Carl Gustav Carus, TU Dresden, Fetscherstr. 74, 01307 Dresden, Germany; 2 Institute for Clinical Genetics, Faculty of Medicine Carl Gustav Carus, TU Dresden, Fetscherstr. 74, 01307 Dresden, Germany; 3 German Cancer Consortium (DKTK), partner site Dresden; German Cancer Research Center (DKFZ), Heidelberg, Germany; 4 National Center for Tumor Diseases (NCT), Dresden, Germany

**Keywords:** Glioma, BIRC5/Survivin, DNA damage, p53, Chromosomal instability

## Abstract

**Background:**

Survivin, belonging to the inhibitor of apoptosis (IAP) gene family, is abundantly expressed in tumors. It has been hypothesized that Survivin facilitates carcinogenesis by inhibition of apoptosis resulting in improved survival of tumorigenic progeny. Additionally, Survivin plays an essential role during mitosis. Together with its molecular partners Aurora B, Borealin and inner centromere protein it secures bipolar chromosome segregation. However, whether increased Survivin levels contribute to progression of tumors by inducing chromosomal instability remains unclear.

**Methods:**

We overexpressed Survivin in U251-MG, SVGp12, U87-MG, HCT116 and p53-deficient U87-MG^shp53^ and HCT116^p53−/−^ cells. The resulting phenotype was investigated by FACS-assisted cell cycle analysis, Western Blot analysis, confocal laser scan microscopy, proliferation assays, spectral karyotyping and in a U251-MG xenograft model using immune-deficient mice.

**Results:**

Overexpression of Survivin affected cells with knockdown of p53, cells harboring mutant p53 and SV40 large T antigen, respectively, resulting in the increase of cell fractions harboring 4n and >4n DNA contents. Increased γH2AX levels, indicative of DNA damage were monitored in all Survivin-transduced cell lines, but only in p53 wild type cells this was accompanied by an attenuated S-phase entry and activation of p21^waf/cip^. Overexpression of Survivin caused a DNA damage response characterized by increased appearance pDNA-PKcs foci in cell nuclei and elevated levels of pATM S1981 and pCHK2 T68. Additionally, evolving structural chromosomal aberrations in U251-MG cells transduced with Survivin indicated a DNA-repair by non-homologous end joining recombination. Subcutaneous transplantation of U251-MG cells overexpressing Survivin and mycN instead of mycN oncogene alone generated tumors with shortened latency and decreased apoptosis. Subsequent SKY-analysis of Survivin/mycN-tumors revealed an increase in structural chromosomal aberrations in cells when compared to mycN-tumors.

**Conclusions:**

Our data suggest that increased Survivin levels promote adaptive evolution of tumors through combining induction of genetic heterogeneity with inhibition of apoptosis.

**Electronic supplementary material:**

The online version of this article (10.1186/s12885-017-3932-y) contains supplementary material, which is available to authorized users.

## Background

Tumors can be considered as dynamic tissues. During disease progression genetic and epigenetic changes lay the foundation for selection and adaptive evolution of cancer cells to survive endogenous stress and to resist environmental challenges [1]. In particular chromosomal instability (CIN) contributes to the development of genetic heterogeneity in tumors and allows the outgrowth of tumorigenic cells with advantageous karyotypes [2]. Survivin, which is found overexpressed in cancer tissues [3], might play a pivotal role in this kind of adaptive evolution of tumors since it exerts dual function in apoptosis and mitosis [4–6]. In gliomas increased levels of Survivin are associated with proliferation markers, histological malignancy grade, and are inversely associated with prognosis [7]. Interestingly, *BIRC5*, the gene encoding Survivin is transcriptionally repressed by p53 [8, 9]. It is therefore conceivable, that loss of functional p53 either due to mutations or its increased mdm2-mediated degradation caused by deregulated signaling pathways in glioblastoma (i.e. loss of PTEN, enhanced PI3K-signaling, for reviewing see [10]) increases a tumor-promoting activity of Survivin.

Originally, due to its baculoviral inhibitor of apoptosis repeat (BIR) domain, Survivin was technically classified as a member of the inhibitor of apoptosis protein (IAP) family [11]. Yet, in contrast to all other IAPs involved in apoptosis inhibition, Survivin lacks a C-terminal RING finger and contains only one BIR [11, 12]. Several studies have demonstrated that overexpression of Survivin protects cells against various apoptotic stimuli such as radiation and chemotherapeutic drugs [13–16]. However, the mechanism of how Survivin inhibits apoptosis has remained obscure. It has been suggested that Survivin directly inhibits caspases [17–20]. Other studies, proposing that Survivin executes its anti-apoptotic function via stabilizing the X-linked IAP (XIAP) or by antagonizing pro-apoptotic smac/DIABLO, also remain controversial [21–24]. In contrast to the aforementioned IAP function, the mitotic role of Survivin is well established [5]. Its mitotic localization is consistent with chromosomal passenger proteins (CPP) Aurora B, Borealin and inner centromere protein (INCENP) [25–27]. During early mitosis, Survivin accumulates at mitotic histone marks at centrosomes introduced by Haspin-mediated phosphorylation of Thr3 on histone 3 (H3) [28–30]. By binding to phosphorylated H3, Survivin then redirects its CPP partners to inner centromeres to form a so-called chromosomal passenger complex (CPC). At this localization, Aurora B kinase, the enzymatic subunit of the CPC, corrects syntelic and merotelic errors in kinetochore-microtubule attachment and therefore guarantees equal sister chromatid distribution [31–33]. When anaphase starts, Survivin and its CPP partners dissociate from kinetochores but in telophase re-aggregate at the polar end of microtubules demarcating the cleavage furrow [16, 34]. At this location, the CPC phosphorylates proteins regulating the contractile actin-myosin ring such as MgcRacGAP and SHC SH2-domain binding protein 1 (SHCBP1) [35, 36]. Cells with blocked function of Survivin or of one of its CPP partners due to chemical inhibitors, RNAi-knockdown or expression of dominant-negative mutants consistently show disturbed chromosomal segregation and defective cytokinesis [15, 16, 31, 37–41].

Up to now, several lines of evidence and our previous studies indicate that also increased expression of CPPs can result in mitotic defects. More specifically, in Chinese hamster CHO cells, human CD34-positive hematopoietic stem cells, human fibroblasts and glioma cells overexpression of Survivin and of Aurora B, respectively, resulted in the appearance of polyploid cell progeny [37, 42–44]. In budding yeast, simultaneous overexpression of Aurora B and INCENP orthologues Ipl1 and Sli15 caused continuous disruption of kinetochore-microtubule attachments leading to tetraploidy [45]. Accordingly, inducible induction of Aurora B in transgenic mice resulted in the development of aneuploidy and multiple spontaneous tumors in mice [46]. Hitherto, it is generally accepted that deregulated Aurora B levels increase the risk for carcinogenesis by induction of CIN, leading to progeny bearing numeric and structural chromosomal aberrations [41, 47, 48]. So far, it has remained largely unclear whether increased levels of Survivin, besides its IAP function, contribute to genetic heterogeneity (i.e. aneuploidy) of tumors. In this study, we sought to investigate a possible role of increased cellular Survivin levels in inducing CIN. Furthermore, it was of special interest whether Survivin-induced CIN enhances tumorigenicity of cancer cells.

Our results show that overexpression of Survivin lead to a significant increase in the fraction of cells experiencing mitotic defects (i.e. lagging chromosomes) and DNA damage in p53 wild type cells (U87-MG, HCT116), SV40 large T antigen (SV40 TAg) expressing cells (SVGp12), mutant p53 cells (U251-MG) and cells with knockdown of p53 (U87-MG^shp53^, HCT116^p53−/−^). In tumor cells expressing p53 wild type, DNA damage was accompanied by induction of a DNA-damage response (DDR) and upregulation of p21^waf/cip^ protein levels. Yet, in p53-deficient U87-MG^shp53^ and HCT116^p53−/−^ cells induction of p21^waf/cip^ was completely abolished and resulted in augmented S-Phase entry of U87-MG^shp53^ glioma cells. Mechanistically the DNA-damage and DDR induced by overexpressed Survivin resulted in non-homologous end joining (NHEJ) repair of chromosomal breaks, indicated by increased chromosomal structural changes, and an increase in aneuploidy as shown by SKY analysis of U251-MG cells.

Interestingly, transduction of Survivin into less-tumorigenic U251-MG cells did not result in efficient tumor formation in immunodeficient mice. Captivatingly, simultaneous overexpression of Survivin and oncogenic mycN generated tumors with shortened tumor latency, decreased apoptosis and displaying an increased frequency of structural chromosomal aberrations when compared to mycN-tumors. In conclusion, our results highlight a possible role of deregulated Survivin protein levels in development of aneuploidy and progression of tumors.

## Methods

### Cell lines

The diploid U87-MG cell line contains p53 wild type alleles and the near triploid U251-MG glioblastoma cell line contains mutant p53R273H [49, 50] and were cultivated as described previously [40]. The human fetal astroglial SVGp12 cell line (ATCC® CRL8621) was generated by immortalization using of SV40 large T antigen (SV40 TAg) as described earlier [51] and cultivated using Minimal Eagle Medium (MEM) Alpha Glutamax (Life Technologies, Darmstadt, Germany) supplemented with 10% *v*/v heat-inactivated FCS (Gibco), 2 mM L-glutamine and 1% non-essential amino acids (Biochrom, Berlin, Germany). The hypodiploid colorectal carcinoma-derived HCT116 cells and HCT116 cells with knockout of p53, designated HCT116^p53−/−^ (kindly provided by B. Vogelstein, Johns Hopkins University, Baltimore) were cultured in RPMI medium (Life Technologies) supplemented with 10% FCS, 2 mM L-glutamine, 10 mM HEPES, 100 U/ml penicillin and 100 μg/ml streptomycin. The human embryonic kidney cells 293 T were maintained in Dulbecco’s modified Eagle medium (DMEM) containing 4.5 g/l glucose (Life Technologies) supplemented with 10% FCS, 10 mM HEPES, 100 U/ml penicillin and 100 μg/ml streptomycin. HCT116, U87-MG, and U251-MG cell lines were authenticated (Multiplexion GmbH, Heidelberg, Germany) and cultured at 37 °C with 5% CO_2_.

### Vectors and transduction of cells

A synthetic cDNA encoding full 142 amino acid Survivin and fused to c-myc and HA tags (Eurofins MWG Biotech) was ligated into appropriate *Age*I and *Not*I restrictions sites of the multiple cloning site of the lentiviral vectors pHATtrick-EGFP and pHATtrick-puroR [52, 53], respectively, resulting in the vectors pHATtrick-Survivin-EGFP and pHATtrick-Survivin-puroR. In pHATtrick-EGFP/puroR vectors transgene expression is driven by an internal SSFV U3 and genetically linked to expression of EGFP and puroR, respectively, by a T2A *Thosea assigna* virus element. Lentiviral particles for transduction of glioma cells and of HCT116 cells were produced by a transient three-vector packaging protocol as described previously [52]. Briefly, 4 × 10^6^ 293 T cells were transfected using polyethylenimine (Polysciences, Warrington, PA), pCD/NL-BH, pczVSV-G, and lentiviral vector, respectively. After 20 h, 293 T cells were incubated with 10 mM sodium butyrate (Sigma-Aldrich, Taufkirchen, Germany) for 6 h. At 24 h after the replacement of sodium butyrate by fresh medium, the lentiviral supernatant was removed from cells and passed through a 0.45 μm filter, mixed with 8 mg/ml Polybrene (Sigma-Aldrich). After determination of infectious units/ml by transducing serially diluted supernatants in 293 T cells, aliquots of viral particles were stored at −80 °C. Target cell lines were transduced at indicated MOIs with EGFP-expressing vectors or puroR-containing vectors, the latter followed by a 24 h treatment with culture medium containing 10 μg/ml puromycin (Takara Clontech). Unless otherwise indicated for all experiments cells were freshly transduced and polyclonal populations were analyzed 72 h after transduction.

For xenograft experiments, an additional retroviral vector encoding human mycN (pWZLneo-mycN) and a mock control (pWZLneo) (kindly provided by C. Beltinger, Department of Pediatrics and Adolescent Medicine, University Centre Ulm, Germany) were packaged and consecutively transduced as described recently [39]. U251-MG cells were transduced with pHATtrick-EGFP and pWZLneo to generate control cells (U251-MG^C/C^), pHATtrick-Survivin-EGFP and pWZLneo to generate cells expressing Survivin (U251-MG^Survivin/C^), pHATtrick-EGFP and pWZLneo-mycN to generate mycN-expressing cells (U251-MG^mycN/C^) and pHATtrick-Survivin-EGFP and pWZLneo-mycN to generate cells co-expressing mycN and Survivin (U251-MG^mycN/Survivin^). Transduction efficiencies using pHATtrick-EGFP and pHATrick-Survivin-EGFP at MOI 20 were routinely in the range of 95% to 99% when measuring EGFP. Cells containing pWZLneo and pWZLneo-mycN, respectively, were additionally selected with G418 (450 μg/ml; Gibco). Experiments with such transduced cells were set up at least 14 days after transduction.

### Western blot analysis

Total cells lysates were prepared and analyzed by immunoblotting as described previously [40]. Membranes were incubated with primary antibodies including polyclonal anti-Survivin (AF886, R&D Systems), monoclonal anti c-myc (R950–25, Invitrogen), monoclonal rabbit anti-p21^waf/cip^ (#2947, Cell Signaling), polyclonal goat anti-p53 (AF1355, R&D Systems), monoclonal rabbit anti-p53 S15 (ab1431, Abcam), monoclonal mouse anti-actin (A2228, Sigma), monoclonal rabbit anti-ATM S1981 (#2152–1, Epitomics), polyclonal rabbit anti-ATM (PC116, Merck, Darmstadt, Germany), monoclonal rabbit anti-CHK2 T68 (#2661, Cell Signaling), polyclonal rabbit anti-Cyclin D1 (sc-753, Santa Cruz), polyclonal rabbit anti-Cyclin E (sc-247, Santa Cruz), and monoclonal mouse anti-γH2AX (05–636, Millipore). Bound antibodies were detected using appropriate secondary antibodies conjugated with HRP (Dako, Hamburg, Germany) as described previously [40].

### Staining of DNA and indirect immunofluorescence analysis

Transduced cells were stained with HBSS-Hoechst 33,342 (0.1 μg/ml, Life Technologies) and appearance of multinucleated nuclei, lagging chromosomes, multipolar metaphases, fragmented nuclei and micronuclei of at least 400 nuclei from each treatment group were examined by fluorescence microscopy (Zeiss Axiovert135, Jena, Germany) using Nikon NIS-Element Imaging Software 4.3 (Nikon, Düsseldorf, Germany). Statistical analysis was performed using Student’s *t*-test.

In other experiments treated cells were stained for Survivin protein (AF886, R&D Systems, Wiesbaden, Germany, 1:120) or DNA damage marker γH2AX (05–636, Millipore) and monoclonal mouse anti-phosphoDNA-PK_CS_ T2609 (Abcam), respectively, as described previously [40, 54] using species-specific secondary fluorochrome-conjugated antibodies (Dianova, Hamburg, Germany). DNA counterstaining was accomplished using HBSS-Hoechst or DAPI. Staining of membranes was done using WGA-Texas Red (10 μg/ml, Life Technologies) as previously described [40]. Stained cells were imaged with a Leica SP5 inverse microscope (Leica, Wetzlar, Germany) using 405 nm, 488 nm, 594 nm lasers and 63× NA1.4 or 40× NA1.25 objective lenses. Image acquisition, shutter, Z-axis position, laser lines, and confocal system were controlled using Leica LAS AF software. γH2AX statistics were calculated after analyzing the average percentage of fluorescence-labeled cells in at least 5 random fields of Survivin- and mock-transduced cells, respectively, representing at least 250 cells per group. All experiments were performed at least three times with similar results. Statistical analysis was performed using Student’s *t*-test.

### Flow cytometry analysis

Propidium iodide (PI) staining analysis was carried out in a MACSQuant flow cytometer as described previously [39]. Analysis of raw data was performed using FlowJo software (Tristar, Inc.). For DNA-synthesis analysis and concomitant cell cycle analysis cells were incubated with 10 μM BrdU (Sigma), stained with PI and analyzed as described previously [39]. FACS data processing includes doublet discrimination and debris exclusion. All experiments were performed at least three times with similar results. Statistical analysis was performed using Student’s *t*-test.

### Cytogenetic analysis

Spectral karyotyping was performed as described previously [40]. Briefly, U251-MG cells were treated with colcemid for 60 min at a concentration of 0.035 μg/ml, incubated in 0.075 M KCl for 20 min at 37 °C, and fixed in a freshly prepared mixture of methanol/acetic acid (3:1) at room temperature. The cell suspension was dropped onto glass slides and used for SKY [40]. SKY images of 15–20 randomly selected metaphase chromosomes per Survivin-transduced cells and mock-transduced cells stained with a mixture of 5 fluorochromes were captured using an DMRXA epifluorescence microscope (Leica GmbH, Wetzlar, Germany), HCX PL SAPO 63×/1.30 oil objective (Leica), SpectraCube® system (Applied Spectral Imaging, Migdal HaEmek, Israel), and analyzed using SKYView® imaging software (Applied Spectral Imaging). Loss or gain of a chromosome was identified by comparing with the karyotype of parental U251-MG cells and designated “off-mode” chromosome. Figure 5d depicts the relative mean of “off-mode” chromosomes in metaphases of Survivin and mock-transduced cells, or in other words the mean percentage of new arising chromosomal gains and losses per cell. New, non-clonal chromosomal breaks and chromosomal aberrations per metaphase caused by overexpression of Survivin or transduction of mock control were also investigated. Statistical analysis was performed with Student’s *t* test. Mann-Whitney-U test was carried out to compare frequencies of structural chromosomal aberrations.

### Animal experiments

Animal experiments were strictly performed in compliance with institutional and state guidelines for the care and protection of animals and were approved by the Local Ethics Committee of the TU Dresden and the Landesdirektion Dresden. A total of 2 × 10^6^ viable U251-MG cells in PBS without additives were subcutaneously injected into the flanks of NMRI-Foxn1^nu^/Foxn1^nu^ mice. Mice were monitored at least twice per week and tumor size was measured. Tumors were measured in two dimensions twice per week, using a digital caliper. Once the tumor exceeded 18 mm in any of the three perpendiculars or animals appeared to be in distress, mice were euthanized. For further experiments and analyses three representative U251-MG^mycN/Survivin^ tumors and U251-MG^mycN/C^ tumors, tumors were excised and cut in two pieces, one for immunohistochemically analysis and the other for the preparation of single cell suspensions. Single cells were obtained by a brain tumor dissociation kit (Miltenyi Biotech GmbH, Bergisch Gladbach, Germany). Afterwards, EGFP-positive cells were sorted using a BD FACS ARIA II cell sorter to exclude contaminating mouse cells and validated for ectopically expressed mycN and mycN/Survivin, respectively. Tumor cells from U251-MG^mycN/Survivin^ tumors #2, #4, #5 and U251-MG^mycN/C^ tumors #16, #17, #19 were subsequently cultured and used for cytogenetic analysis. 2 × 10^6^ sorted cells from tumors #2 and #19 and additionally freshly transduced U251-MG^mycN/Survivin^ and U251-MG^mycN/C^ cells, respectively were used for subcutaneous transplantation (eight mice per group). Statistical analysis of tumor growth and survival were performed with Student’s *t* test and by log rank test, respectively.

### Ex vivo culture of sorted tumor cells and soft agar assay

Sorted EGFP-positive cells from tumors were cultured as described above. After two weeks developing tumor cell spheroids were photographed and cell aggregates exceeding 100 μm in diameter were quantified. Soft agar assays were performed as described recently [55]. Briefly, a 3% solution of agar (56 °C) was diluted to a final concentration of 0.6% with growth medium (at 56 °C), pipetted into tissue culture dishes, and allowed to solidify at room temperature. 1 × 10^4^ prepared tumor cells were adjusted to a volume of 50 μl and were mixed with 0.3% (diluted with growth medium at 40 °C), pipetted gently onto the bottom agar layer, and allowed to solidify for 30 min at room temperature. This was done in triplicates per tumor. Then the cells were incubated at 37 °C in a 5% CO_2_ atmosphere and fed twice per week. After 2 weeks in culture, colonies that had formed within the soft agar were stained with crystal violet (0.2 mg/ml MTT, Sigma) in ddH_2_O. Colonies with a minimal size of 100 μm were counted and the area was calculated using the ImageJ plugin “Colony counter”. Statistical analysis of colony numbers was performed with Student’s *t* test.

### Immunohistological analysis

Cryoslices were used for analyzing apoptosis and proliferation. Analysis of apoptosis in tumors was performed using TUNEL (in situ cell death detection kit, Roche, Mannheim, Germany) and analysis of proliferation was done using Ki67 antibodies (Leica, Biosystems, Wetzlar, Germany) using the protocols recommended by the suppliers. DNA counterstaining was accomplished with Mayer’s hemalaun solution. The apoptosis index and Ki67-proliferation index were calculated by analyzing the average percentage of labeled cells in at least 10 random fields from different sections at × 400 magnification. Statistical analysis was performed using Student’s *t*-test.

## Results

### Overexpression of Survivin leads to an increase of cells with mitotic defects

**Fig. 1:**
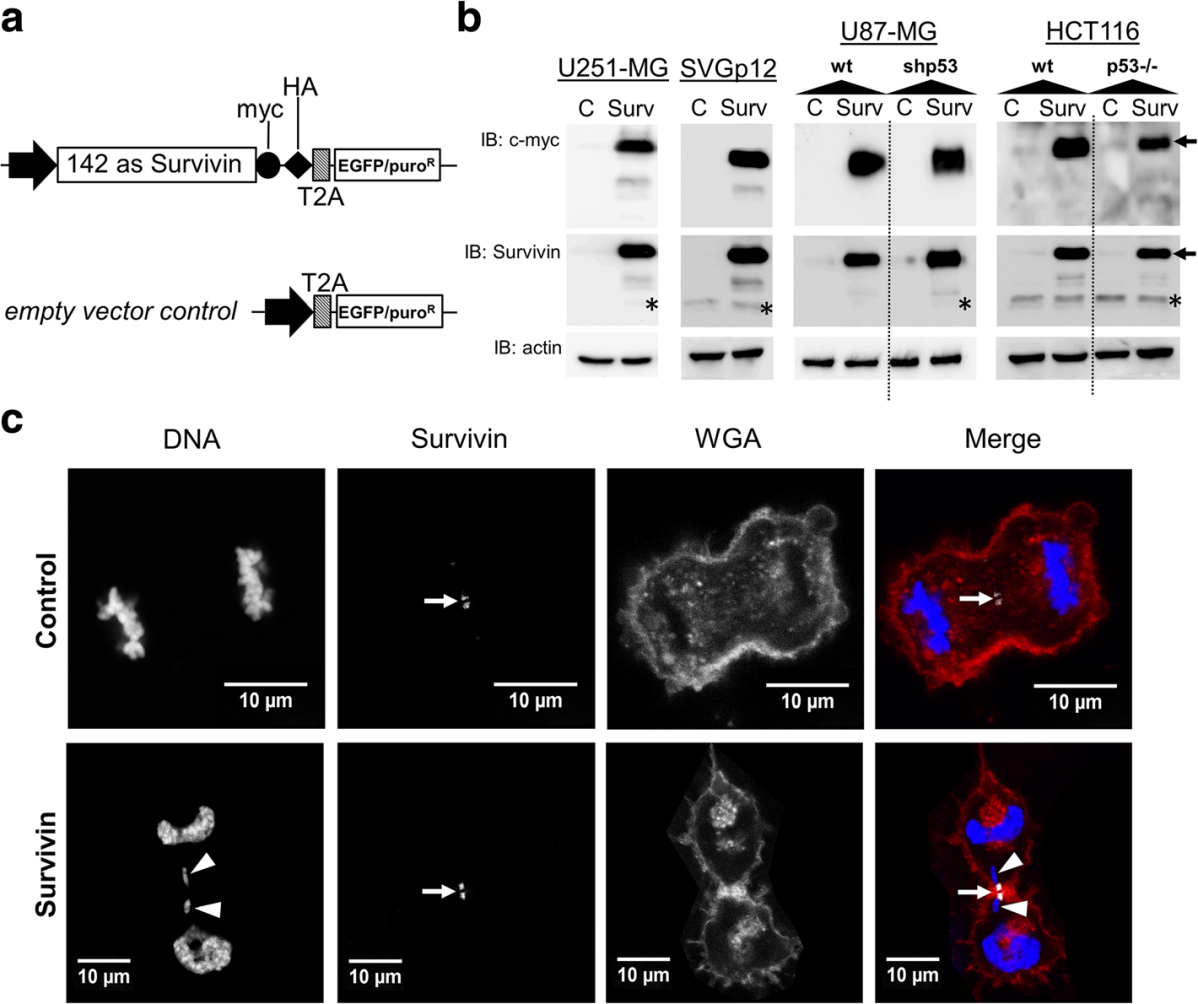
Ectopic expression of Survivin in different cell lines. **a** Scheme of the Survivin (top) and control (bottom) constructs including the coding sequence for Survivin fused to c-myc and HA tags. T2A: self-cleaving 2A peptide; puroR: puromycin resistance. **b** Western blot analysis demonstrating ectopic Survivin in U251-MG, SVGp12, U87-MG, U87-MG^shp53^, HCT116 and HCT116^p53−/−^ cells. As control empty vector-transduced cells were used. Cells were either transduced with lentiviral pHATrick-Survivin-EGFP (Surv) or empty vectors (C) and lysed 72 h after transduction. Ectopic Survivin (24 kDa) (black arrows) and endogenous Survivin (16.5 kDa) (asterisks) bands are indicated. The membranes were re-probed with antibodies specific for α-actin (42 kDa) to confirm equal loading. **c** Indirect immunofluorescence analysis using specific anti-Survivin antibodies revealed typical immunosignals in for endogenous Survivin and for Survivin-transduced cells at the cleavage furrow and midbodies (arrows). Note the damaged lagging chromosome which seems broken after cytokinesis (white arrowheads). From left to right: DNA (DAPI), Survivin (FITC), membrane (WGA-TexasRed) and merge

In order to corroborate whether increased levels of Survivin affects mitosis and eventually leads to chromosomal instability, we generated lentiviral vectors for expression of the 142 amino acid (as) coding sequence of Survivin fused to c-myc and HA-tags and used the empty vector (mock) as control (Fig. 1a).

In initial experiments we tested different MOIs of Survivin-vector and empty vector to address a possible induction of mitotic defects in U251-MG and U87-MG^shp53^ cells and to exclude adverse effects of increasing viral titers. Increasing viral titers to 50 MOI severely affected survival of cells transduced with Survivin and empty vector (data not shown). Therefore, in our subsequent experiments we routinely used 20 MOI for transduction of cells.

**Fig. 2:**
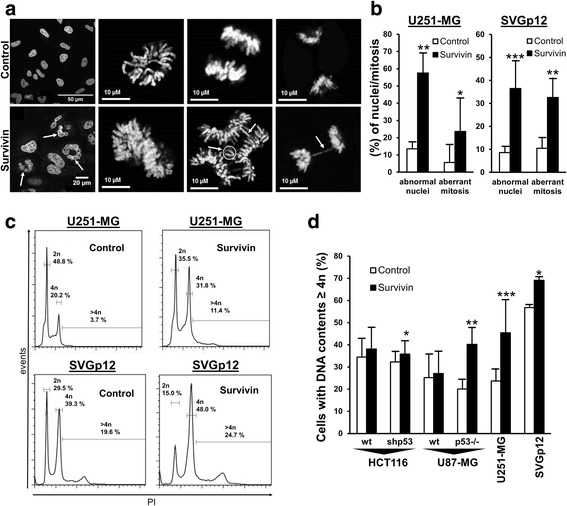
Forced expression of Survivin affetcs mitosis and augments polyploidy in cells with mutant p53, loss of p53 and astrocytoma cells with expression of SV40 TAg. **a** Representative images of U251-MG cells stained with DAPI (chromosomes) (top panel: control cells; bottom panel: Survivin cells). Abnormal interphase cells (white arrows) and increased chromosome numbers as well as lagging chromosomes are depicted (white circle and white arrows). **b** Relative mean percentages of U251-MG and SVGp12 cells displaying abnormal nuclei and aberrant mitoses (mean values ± SD; *p < 0.051**. *p* < 0.01, ***p < 0.001; Student’s *t*-test. **c** Representative FACS-assisted DNA-content analysis of U251-MG (top panel) and SVGp12 cells (bottom panel) with overexpression of Survivin compared to the empty vector transduced controls. Note the increased 4n peak and >4n populations. **d** Mean increase in the fraction of Survivin-transduced cells with DNA content of 4n and >4n compared to empty vector-transduced controls (mean values ± SEM). Cell lines with loss of p53 function showed increased cell fractions with DNA content ≥4n in contrast to cells containing wildtype p53. *p < 0.05, **p < 0.005, ***p < 0.001. All data were collected 72 h after transduction of cells

As depicted in Fig. 1b transduction of Survivin-myc-HA at 20 MOI resulted in a robust expression of the transgene in U251-MG, SVGp12, wild type and p53-deficient U87-MG and HCT116 cells, respectively. Confocal laser scan analysis of Survivin-tranduced U251-MG glioblastoma cells and of SVGp12 immortalized astroglial cells revealed an increased appearance of cells with lagging chromosomes, increased chromosome numbers, or irregular metaphases. Yet, staining cells with an antibody specific for Survivin revealed typical immunosignals for Survivin at midbodies and at kinetochores (Fig. 1c and Additional file 1: Figure S1). Additional quantitative analysis of DAPI-stained nuclei in transduced U251-MG and SVGp12 cells confirmed a significant increase in the relative fraction of cells containing abnormal nuclei (i.e. multiple nuclei or huge polymorphic nucleus per cell) and a significant increase in the relative fraction of aberrant mitoses (i.e. non-aligned chromosomes in metaphase, increased chromosome numbers, lagging chromosomes) (Fig. 2a, b). FACS-assisted DNA-content analysis of cells showed background levels of polyploidy in the analyzed cancer cell lines. However, ectopic overexpression of Survivin further increased cell fractions with DNA content of 4n and 8n (for representative examples see Fig. 2c) suggesting induction of tetraploid/octaploid progeny in cells containing mutant p53 (U251-MG), deleted p53 (U87-MG^shp53^, HCT116^p53−/−^) or SV40 TAg (SVGp12). In contrast, in U87-MG and HCT116 cells harboring wild type p53, increased tetraploidy/polyploidy was not induced by overexpression of Survivin (Fig. 2d).

### Forced expression of Survivin results in DNA-damage and differentially affects cell cycle of p53 wildtype cells and p53-deficient glioma cells

**Fig. 3:**
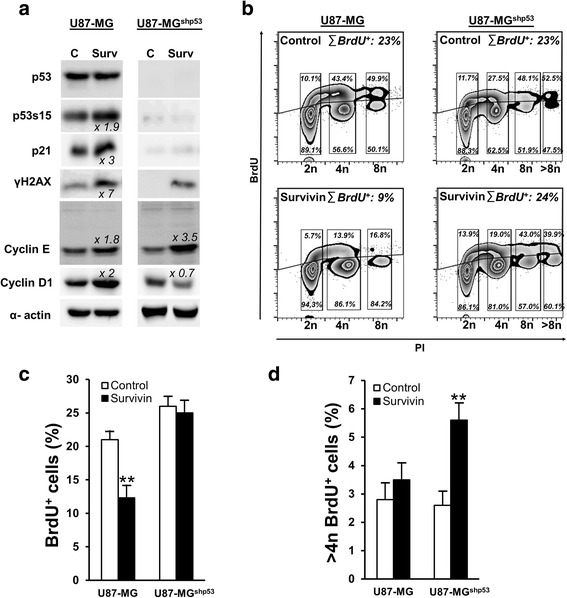
Overexpression of Survivin in glioma cells affects cell cycle and proliferation in a p53 dependent manner. **a** Western blot analysis showing cell lysates from U87-MG and U87-MG^shp53^ after transduction with Survivin and control vectors. The membranes were blotted with anti-p53 (53 kDa), anti-p53s15 (53 kDa), anti-p21^waf/cip^ (21 kDa), anti-γH2AX (16 kDa), anti-cyclin D1 (37 kDa) and E (42 kDa singlets +50 kDa doublets) antibodies and the signals comparing control and Survivin cells were measured. The relative band density (fold increase) obtained from the densitometric analysis and normalized to the corresponding control is depicted. α-actin (42 kDa) was used as an internal loading control. **b** Representative BrdU-incorporation assays of U87-MG (left panel) and U87-MGs^hp53^ cells (right panel) with overexpression of Survivin (bottom) compared to the empty vector-transduced controls (top). The overall amount of BrdU-positive fractions as well as the relative levels of BrdU^+^ and BrdU^−^ cells in fractions with DNA contents of 2n, 4n, 8n and >8n are depicted. **c** Proliferation index of the U87-MG and U87-MG^shp53^ cells transduced with Survivin and empty vector controls. Depicted are mean values ± SD. **p < 0.01. **d** BrdU-incorporation in U87-MG and U87-MG^shp53^ cell fractions with DNA content >4n. Depicted are mean values ± SD. **p < 0.01. All data were collected 72 h after transduction of cells

To address how p53 status influences the development of mitotic defects after enforced expression of ectopic Survivin, and to exclude a possible G2/M-arrest leading to increased numbers of tetraploid cells, we analyzed the cell cycle of U87-MG wild type cells harboring normal p53 and U87-MG^shp53^ cells with stable RNAi-mediated knockdown of p53 by Western blot analyses and fluorescence cytometry. In U87-MG wild type cells increased expression of Survivin resulted in phosphorylation of p53(S15) and in an induction of CDK4/6 inhibitor p21^waf/cip^. Together with a concomitant increase in Cyclin D1 and Cyclin E protein steady state levels this hints to a p21^waf/cip^-mediated G_1_ cell cycle arrest of U87-MG cells (Fig. 3a) but no G2/M-arrest. Yet, most likely due to the loss of p53 and absence of its transcriptional target p21^waf/cip^, such a cell cycle arrest was not induced in U87-MG^shp53^ cells (Fig. 3a), resulting in faster progression into S-phase characterized by decrease in cyclin D1 levels. However, an increase in Cyclin E levels likely indicated a prolonged S-phase entry in U87-MG^shp53^ cells transduced with Survivin when compared to mock-transduced U87-MG^shp53^ cells and also to Survivin-transduced U87-MG cells. This might account to additional time needed for building pre-replication complexes for DNA synthesis in tetraploid/polyploid cells.

When referring to FACS-assisted cell cycle analysis it became obvious that the observed induction of p21^waf/cip^ in U87-MG wild type cells after overexpression of Survivin led to a decrease in BrdU-positive cells, indicating a transient G_1_ arrest and subsequent attenuated S-phase entry when compared to cells transduced with empty vectors (Fig. 3b, c). More specifically, when analyzing the relative BrdU-incorporation levels in 2n, 4n and 8n fractions of these cells it became obvious that with increasing DNA contents less cells entered S-phase (Fig. 3b). Such an effect was not observed in U87-MGshp53 cells transduced with Survivin which is likely due to the fact that these cells are devoid of p53 and therefore cannot restrict G1/S-transition (Fig. 3b,c). Instead, U87-MG^shp53^ cells overexpressing Survivin were able to incorporated BrdU at DNA contents of 8n and >8n which indicates ongoing endoreplication of the genome (Fig. 3b, d). BrdU incorporation in cells having DNA contents >4n were also observed in U251-MG and SVGp12 cells after ectopic overexpression of Survivin (Additional file 2: Figure S2a, b). Collectively, this shows that the development of tetraploid (4n) and even polyploid cell fractions can be induced by deregulated Survivin levels and is facilitated by loss of p53 function.

**Fig. 4:**
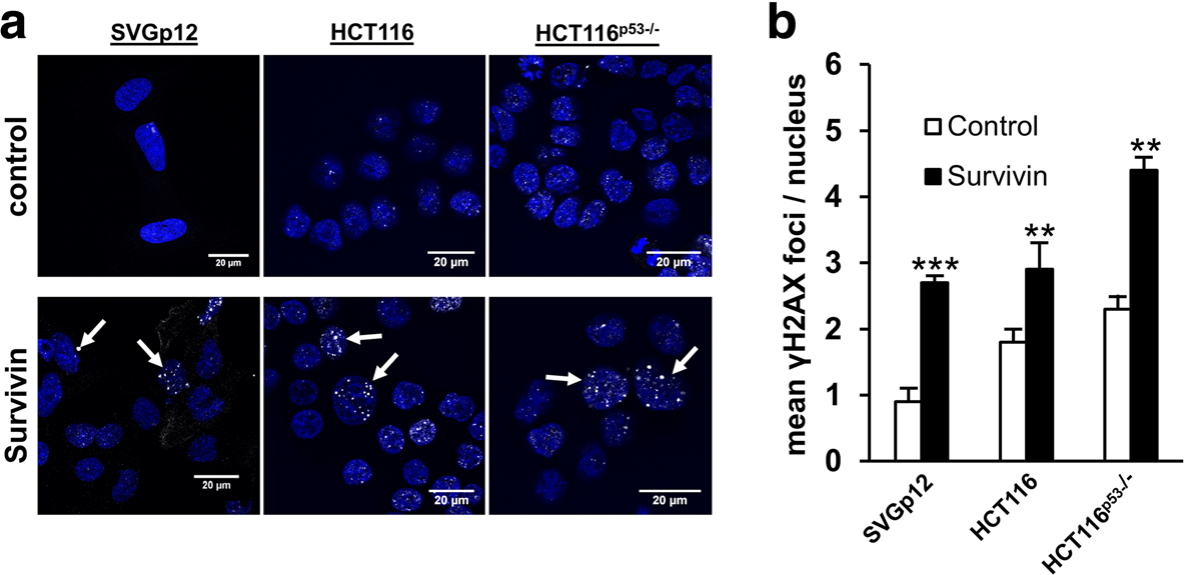
Overexpression of Survivin causes DNA damage. **a** Indirect immunofluorescence analysis showing increased numbers of γH2AX foci in SVGp12, HCT116 and HCT116^p53−/−^ cells 72 h after transduction with Survivin. γH2AX foci are marked with arrows. Upper panel: empty vector-transduced controls; lower panel: Survivin-transduced cells. DAPI (DNA, blue), FITC (γH2AX, white foci). **b** Graph depicting the mean number of foci and SEM per cell in each cell line. **p < 0.01; ***p < 0.001

Interestingly, overexpression of Survivin in U87-MG as well as U87-MG^shp53^ cells caused increased protein levels of γH2AX, a marker for DNA-double strand breaks (DSB), which is linked to the observed p53-dependent cell cycle arrest and furthermore might be related to Survivin’s CPP function (Fig. 3a). To confirm these results, experiments were recapitulated using the colon carcinoma cell line HCT116 and its isogenic p53-knockout cell line HCT116^p53−/−^. As expected, overexpression of Survivin in those cells resulted in increased γH2AX expression as well as a p53-dependent induction of p21^waf/cip^ when compared to mock-controls (Additional file 3: Figure S3). The induction of γH2AX was also confirmed by indirect immunofluorescence analysis in SVGp12, HCT116, and HCT116^p53−/−^ cells (Fig. 4a). After transduction of Survivin, γH2AX foci were monitored in nuclei of cells in a stochastic pattern, whereas fewer signals were detected in cells transduced with the mock control. In general, overexpression of Survivin resulted, depending on the cell line, in the mean in 3 to 4 γH2AX foci per nucleus indicating a moderate DNA damage (Fig. 4b).

### Overexpression of Survivin in glioma cells induces chromosomal instability

**Fig. 5:**
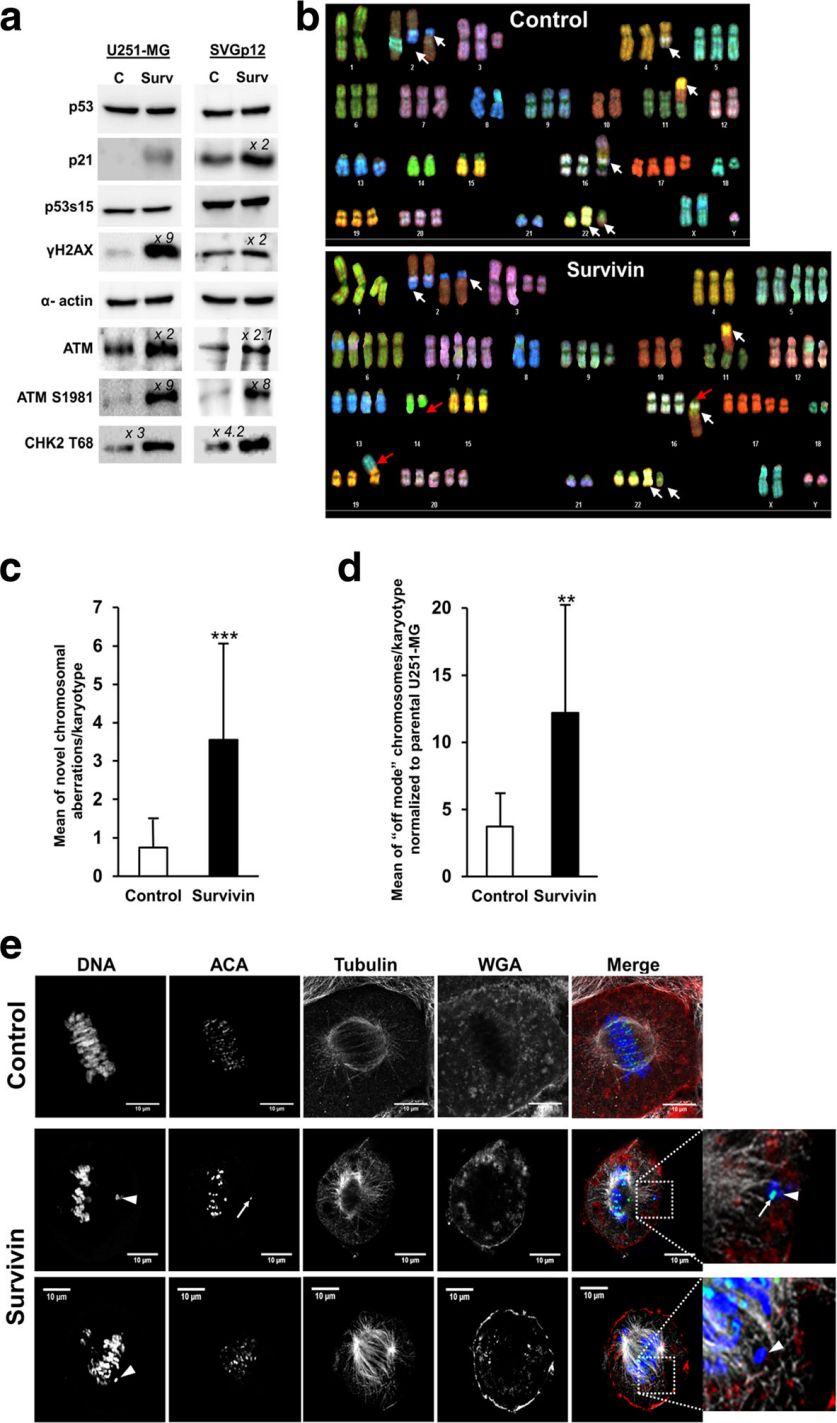
DNA-damage response and induction of aneuploidy in Survivin-transduced glioma cells. **a** Western Blot analysis showing the activation of a DNA damage response and a stabilized p53 protein in U251-MG and SVGp12 cells that overexpress Survivin. The membranes were blotted with anti-p53 (53 kDa), anti-p21^waf/cip^ (21 kDa), anti-p53s15 (53 kDa), anti-γH2AX (16 kDa), anti-ATM (350 KDa), anti-pATM S1981 (350 kDa) and anti-pCHK2 T68 (62 kDa) antibodies. The indicated values represent the relative band density (fold increase) obtained by densitometric analysis when compared to the empty vector-transduced control. Membranes were re-probed with α-actin (42 kDa) to confirm equal loading. **b**, **c**, **d** SKY-Analysis reveals chromosomal instability (increased numerical and structural chromosomal aberrations) in Survivin-overexpressing U251-MG cells compared to mock-control. **b** shows representative karyograms of mock-control (upper figure) and Survivin-overexpressing cells (lower figure) with white arrows indicating clonal aberrations already present in the parental cell line. Survivin-overexpressing U251-MG show additional non-clonal structural changes indicated by purple arrows and also increased ploidy. The number of such non-clonal structural aberrations per metaphase was significantly increased compared to mock-control (**c**). Additionally, when counting gains and losses of chromosomes per chromosome and metaphase (compared to the mean number of a particular chromosome in the parental cell line), we found a significant higher number of “off-mode” chromosome numbers (**d**). **p < 0.001, ***p < 0.0005. **e** Indirect immunofluorescence analysis of U251-MG cells transduced with empty vector (Control) or with Survivin-vector. Note the non-aligned chromosomes (white arrowheads) in Survivin-transduced cells either containing or lacking immunosignals for centromers (white arrows). All data were collected 72 h after transduction of cells

Since it was of special interest whether a DNA-damage response (DDR) occurs after overexpression of Survivin, we analyzed the protein levels of activated DNA-damage sensor kinase ATM in U251-MG glioblastoma cells and in SV40 TAg-immortalized human SVGp12 astrocytes. As expected, overexpression of Survivin resulted in induction of γH2AX, phosphorylation of ATM at position S1981 and of its downstream target CHK2 at position T68 (Fig. 5a). In further experiments we noted the increased appearance of phosphoDNA-PKcs foci in nuclei of Survivin-transduced SVGp12 and U251-MG cells (Additional file 4: Figure S4). DNA-PKcs is the catalytic subunit of DNA-PKs/Ku-complex, is a key enzyme involved in the re-ligation of double-stranded DNA breaks.

In SVGp12 and U251-MG cells, p53 protein is stabilized by either by R273H mutation or complexation with SV40 TAg [51, 56]. In line with this, p53 protein was detected at similar levels in Survivin- as well as in mock-transduced cells. Interestingly, an increased expression of p21^waf/cip^ was detected in both cell lines upon overexpression of Survivin, suggesting some residual transactivation capacity described for p53R273H protein [57] and transactivation by p53 wild type protein in SVGp12 cells albeit the presence of SV40 TAg (Fig. 5a).

So far, our studies revealed that overexpression of Survivin lead to increase of DNA contents in p53-deficient cells and a p53-independent moderate DNA damage. Whether these effects cause chromosomal instability leading to aneuploid progeny was investigated using spectral karyotyping (SKY) of U251-MG cells. In order to proper quantify chromosome aberrations we normalized the data obtained from transduced cells against the karyotypes of parental non-transduced U251-MG cells. As depicted in representative SKY analyses in Fig. 5b, transduction with a lentiviral vector encoding Survivin resulted in numerical chromosomal aberrations and an increased number of novel, non-clonal structural chromosomal aberrations not present in parental U251-MG. The frequency of novel chromosomal aberrations was significant higher in Survivin overexpressing cells than in mock-transduced cells (mean structural chromosomal aberrations/Survivin-transduced cell 3.55 ± 2.50 SD; mean structural chromosomal aberrations/mock-transduced cell 0.73 ± 0.75 SD, *p* < 0.0005) (Fig. 5c). The increase in the frequency of chromosomal aberrations in Survivin-overexpressing cells was accompanied with increased gains and losses of chromosomes (“off-mode chromosomes”) in individual karyotypes when compared to empty vector-transduced control. Karyotypes of Survivin-transduced cells contained in the mean more than 3-fold greater off-mode chromosome numbers (gain or loss) than empty vector-transduced cells (both normalized to the karyotypes of parental U251-MG cells, *p* < 0.001) (Fig. 5d). Mechanistically, gain of chromosomes might be linked to false kinetochore-microtubule connections (i.e. syntelic chromosomes) or abortive cytokinesis in Survivin-overexpressing cells. Accordingly, it appeared conceivable that loss of chromosomes observed in Survivin-transduced U251-MG cells is linked to loss of centromeric regions. This notion is supported by confocal laser scanning analysis of Survivin-transduced U251-MG cells, which revealed non-aligned chromosomes in metaphase lacking immunosignals for centromeres when stained with anti-centromere antibodies (Fig. 5e).

Collectively, these results shows, that overexpressed Survivin affects chromosomal stability by disturbing sister chromatid distribution in daughter cells, eventually resulting in DNA-breaks and NHEJ repair of chromosomes.

### Survivin cooperatively increases tumorigenicity of mycN-induced U251-MG xenografts

Initial experiments to assess the impact of overexpressed Survivin in glioma xenografts were performed with U251-MG cells, which, due to an inefficient angiogenesis are less tumorigenic when transplanted into nude mice [58, 59]. We presumed that overexpression of Survivin in U251-MG might be sufficient to promote tumor growth. Yet, U251-MG cells transduced with pHATtrick-Survivin-EGFP as well as transduced with the empty vector did not form substantial tumors after 80 days (data not shown). Therefore, overexpression of Survivin alone was not sufficient to increase tumorigenicity of U251-MG cells. Recently, oncogenic myc as well as mycN was reported to induce a pseudohypoxic glycolysis of glioblastoma cells, which in turn should be less dependent on neoangiogenesis [60]. In order to bypass the inefficient angiogenesis of U251-MG and to furthermore provide increased oncogenic signaling, we serially transduced cells with oncogenic mycN and Survivin generating U251-MG^mycN/Survivin^ cells. As outlined in the material and methods section we also generate mycN-expressing U251-MG (U251-MG^mycN/C^), Survivin-expressing (U251-MG^Survivin/C^) and U251-MG control cells (U251-MG^C/C^).

**Fig. 6:**
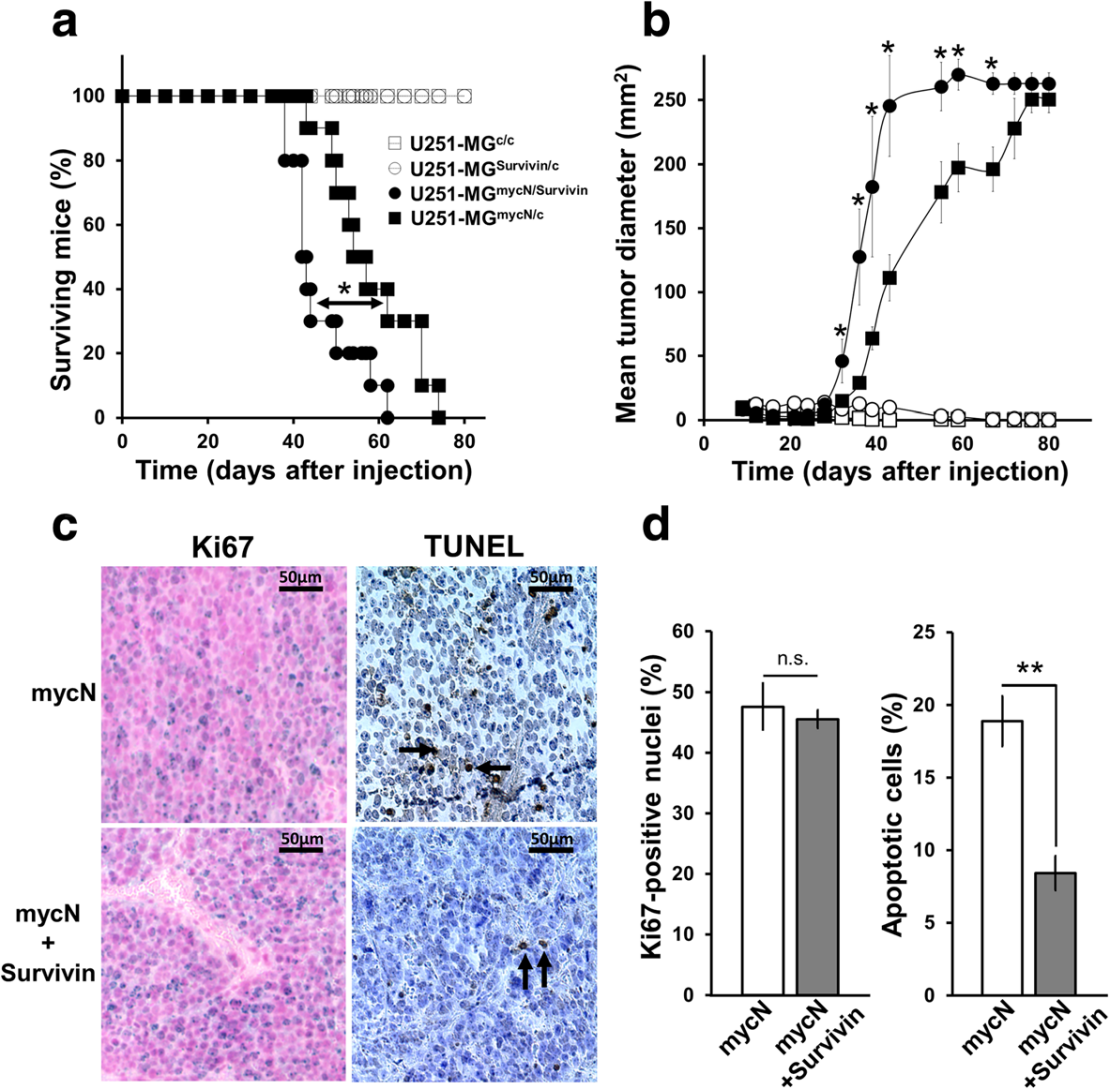
Decreased latency and enhanced growth of U251-MG tumors co-expressing Survivin and mycN. **a** Kaplan Meier survial plot of mice xenografted with indicated differentially transduced U251-MG cells. U251-MG cells harboring simultaneous overexpression of Survivin and mycN generated tumors with significantly shorter latency than cells that only expressed oncogenic mycN. *p < 0.05. **b** Tumor growth curve: tumors co-expressing Survivin and mycN grew significantly faster than tumors containing mycN alone. *p < 0.05. **c** Paraffin-embedded tissue sections were analyzed for proliferation (Ki67) and for apoptosis (TUNEL assay). **d** Quantification of Ki67 positive cells and of apoptotic cells. Survivin/mycN tumors did not exhibit differences in cell proliferation compared to mycN tumors. Note that, tumors co-expressing the transgenes showed markedly decreased apoptosis. Depicted are mean values ± SD. **p < 0.01

In line with our aforementioned results, U251-MG^Survivin/C^ (overexpressing only the Survivin transgene) and U251-MG^C/C^ control cells were not able to efficiently form tumors when transplanted in nude mice. On the other hand, U251-MG^mycN/Survivin^ and U251-MG^mycN/C^ cells efficiently produced subcutaneous tumors. Intriguingly, simultaneous overexpression of Survivin and oncogenic mycN generated tumors with shortened tumor latency and significantly accelerated tumor growth, resulting in decreased survival time of mice when compared to U251-MG^mycN/C^ tumors (Fig. 6a, b). In detail, the mean survival of mice with U251-MG^mycN/Survivin^ tumors was 45 days whereas the mean survival time of mice xenografted with U251-MG^mycN/C^ cells was 58 days (*p* < 0.05).

**Fig. 7:**
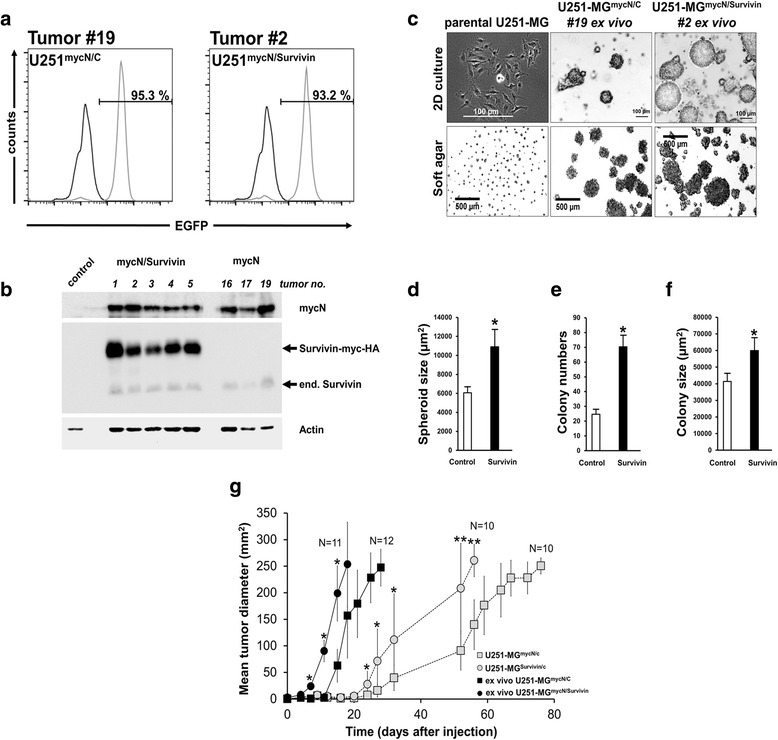
Increased tumorigenicity of mycN-induced U251-MG xenografts. **a** Flow cytometry analysis showing purity of sorted EGFP-positive U251-MG cells (grey line) from tumors #2 and #19. U251-MG wildtype cells were used as negative control (black line). **b** Western blot analysis confirmed expression of transgenic Survivin (24 kDa), endogenous Survivin (16.5 kDa) and mycN (67 kDa) in mycN/Survivin containing tumors #1, #2, #3#, #4, #5 and mycN-containing tumors #16, #17, #19. As additional control non-cancerous mouse tissue (control) was prepared and included in the analysis. Membranes were re-probed with α-actin (42 kDa) to confirm equal loading. **c** U251-MG xenograft-derived cells tranduced with mycN and Survivin and mycN alone form spheres when using standard cell culture conditions. Depicted are the mean values ± SEM. *p < 0.05. **d** Note that U251^mycN/Survivin^ cells formed significantly larger spheres when comparted to U251^mycN/C^ cells. **e**, **f** U251-MG^mycN/Survivin^ cells form more and larger colonies in soft agar than U251-MG^mycN/C^ cells. Depicted are the mean values ± SEM. *p < 0.05 **g** Tumor growth curve re-transplantated U251-MG^mycN/Survivin^ cells and U251-MG^mycN/C^ cells Tumor growth rates are faster when compared to tumors produced by freshly transduced U251-MG^mycN/Survivin^ and U251-MG^mycN/C^ cells. Depicted are the mean values ± SEM. *p < 0.05; **p < 0.01

To determine the mechanisms by which the combined overexpression of Survivin and mycN leads to a more aggressive tumor growth in vivo, slices of tumors were used to analyze apoptosis by TUNEL and cell proliferation by staining with the marker Ki-67, respectively (Fig. 6c, d). Five out of eight U251-MG^mycN/Survivin^ tumors and three out of eight U251-MG^mycN/C^ tumors were selected with respect to a comparable tumor volume to avoid confounding effects of different tumor size (data not shown). The proliferation index of U251-MG^mycN/Survivin^ tumors determined by Ki67-staining was similar to U251-MG^mycN/C^ tumors (Fig. 6c, d) and showed a hyperproliferation of tumors. Interestingly, the co-expression of Survivin and mycN in U251-MG tumors lead to a significant decreased apoptosis when compared to tumors expressing mycN alone (Fig. 6c, d). For subsequent experiments, U251-MG^mycN/Survivin^ and U251-MG^mycN/C^ cells, respectively, were prepared from the same tumors as used for the immunohistological analysis and sorted for EGFP-positive cells (Fig. 7a). Of note, sorted cells still expressed the human transgenes (Fig. 7b). Intriguingly, all ex vivo cultivated U251-MG^mycN/Survivin^ and U251-MG^mycN/C^ cells from the different tumors exhibited an anchorage independent growth and formed spheroids using the standard 2D cell culture protocol and colonies when grown in soft agar (Fig. 7c). However, U251-MG^mycN/Survivin^ cells formed significantly bigger spheroids in 2D culture and significantly more and bigger colonies in soft agar when compared to U251-MG^mycN/C^ cells as depicted for cells from tumor #2 containing mycN and Survivin transgenes and from tumor #19 containing mycN alone (Fig. 7d–f). Similar results were obtained using ex vivo cultivated U251-MG^mycN/Survivin^ and U251-MG^mycN/C^ cells from tumors #4, #5, #16, and #17 (data not shown).

When cells from U251-MG^mycN/Survivin^ tumors and from U251-MG^mycN/C^ tumors were further analyzed by SKY it became evident that cells from tumors co-expressing the Survivin and mycN transgenes contained significant more novel structural chromosomal aberrations (mean 1.333 ± 1.00 SD) than cells from tumors only expressing mycN (mean 0.5 ± 0.61 SD, *p* < 0.005) (for representative SKY-analyses see Additional file 5: Figure S5). Of note, re-transplantation of cells from tumor #2 containing mycN and Survivin transgenes and from tumor #19 containing the mycN transgene alone showed dramatically shortened tumor latency when compared to tumors produced by freshly transduced U251-MG^mycN/Survivin^ and U251-MG^mycN/C^ cells (Fig. 7g). Yet, re-transplanted U251-MG^mycN/Survivin^ tumor cells with co-expression of Survivin and mycN still showed significant decreased tumor latency than re-transplanted U251-MG^mycN/C^ tumor cells expressing mycN transgene alone.

Taken together, our data shows that overexpression of Survivin increases mycN-induced tumorigenicity of U251-MG glioma cells probably by combined induction of CIN and prevention of apoptosis.

## Discussion

In gliomas increased levels of Survivin are linked to tumor aggressiveness, chemoresistance and radioresistance [61–63]. So far, the role of Survivin in tumor progression and resistance still remains ambiguous and is mostly considered to depend on its IAP function. In accordance, ectopic overexpression of Survivin in the skin of transgenic mice has been shown to confer cellular resistance to UVB light and the IAP function of Survivin was suggested to promote the outgrowth of papillomas with mutated p53 tumor suppressor gene [64]. Likewise, the IAP function of Survivin was proposed to facilitate the progression from chemically induced papilloma to squamous cell carcinoma [65] and to initiate hematological malignancies in GATA1-Sur transgenic mice treated with DNA-alkylating N-ethyl-nitrosourea [66]. Similarly, transgenic expression of Survivin in the urinary bladder of transgenic mice increased susceptibility to tumor initiating N-butyl-N-(4-hydroxybutyl) nitrosamine [67]. Moreover, a recent report described less apoptosis in tumors and decreased survival of mice transplanted with immortalized embryonic rat fibroblast transduced with tumor-promoting mycN and Survivin when compared to mice with transplanted tumors genetically engineered only with Survivin or mycN [68]. Noteworthy, whether an induction of chromosomal instability caused by deregulated Survivin levels contributed to tumor initiation or progression through induction of CIN and outgrowth of favorable karyotypes was not addressed in these studies.

In recent comprehensive studies we have elaborated that knockdown of Survivin in permanent as well as primary glioma cell lines had no impact on caspase-dependent apoptosis but instead lead to immense cellular polyploidy with cells having DNA contents up to 32n, poly-merotelic kinetochore-microtubuli connections, DNA damage, DNA damage response, and NHEJ [39, 40]. The DNA damage in p53 wild type cells with knockdown of Survivin was accompanied by a transient G_1_ cell cycle arrest which was not able to halt endoreplication of DNA. Finally, endoreplication of DNA resulted in mitotic catastrophe of cells independently of p53 status [39, 40]. In this study we demonstrate, that the phenotype of glioma cells with ectopic overexpression of Survivin (i.e. G_1_ cell cycle arrest in p53 wild type cells, DNA damage, DNA damage response, and NHEJ) resembles to some extent the Survivin knockdown phenotype described in our previous work [40] and is mechanistically linked to the CPP function of Survivin. Yet, in contrast to the Survivin-RNAi phenotype, overexpression of Survivin did not result in excessive endoreplication of the genome or in mitotic catastrophe. Overexpression of Survivin in cells containing mutant p53 (U251-MG), deleted p53 (U87-MG^shp53^, HCT116^p53−/−^) or SV40 TAg-bound p53 (SVGp12) only lead to a moderate increase in the fraction of cells displaying tetraploidy/polyploidy whereas cells containing wild type p53 were less affected. Interestingly, a previous study also demonstrated an increase in murine hematopoietic stem cells with DNA contents >4n after transduction of wild type Survivin which was further accelerated in cells with knockout of the CDK4/6 inhibitor p21^waf/cip1^ [69]. Likewise, increased protein levels of Survivin after knockout of Cullin 9, a scaffold component of E3 ubiquitin ligases marking Survivin and possibly other mitotic proteins for proteolytic degradation, resulted in increased polyploidy in mouse embryonic fibroblasts (MEFs) which was dramatically enhanced by co-deletion of p53 [70]. Collectively, these results show that loss of p53 and of its transcriptional target p21^waf/cip1^ facilitate the development of polyploidy in cells following deregulated expression of Survivin. In line with a previous study, our data confirm that a functional p53/p21^waf/cip1^ axis attenuates cell cycling and outgrowth of aneuploid cells [71].

**Fig. 8:**
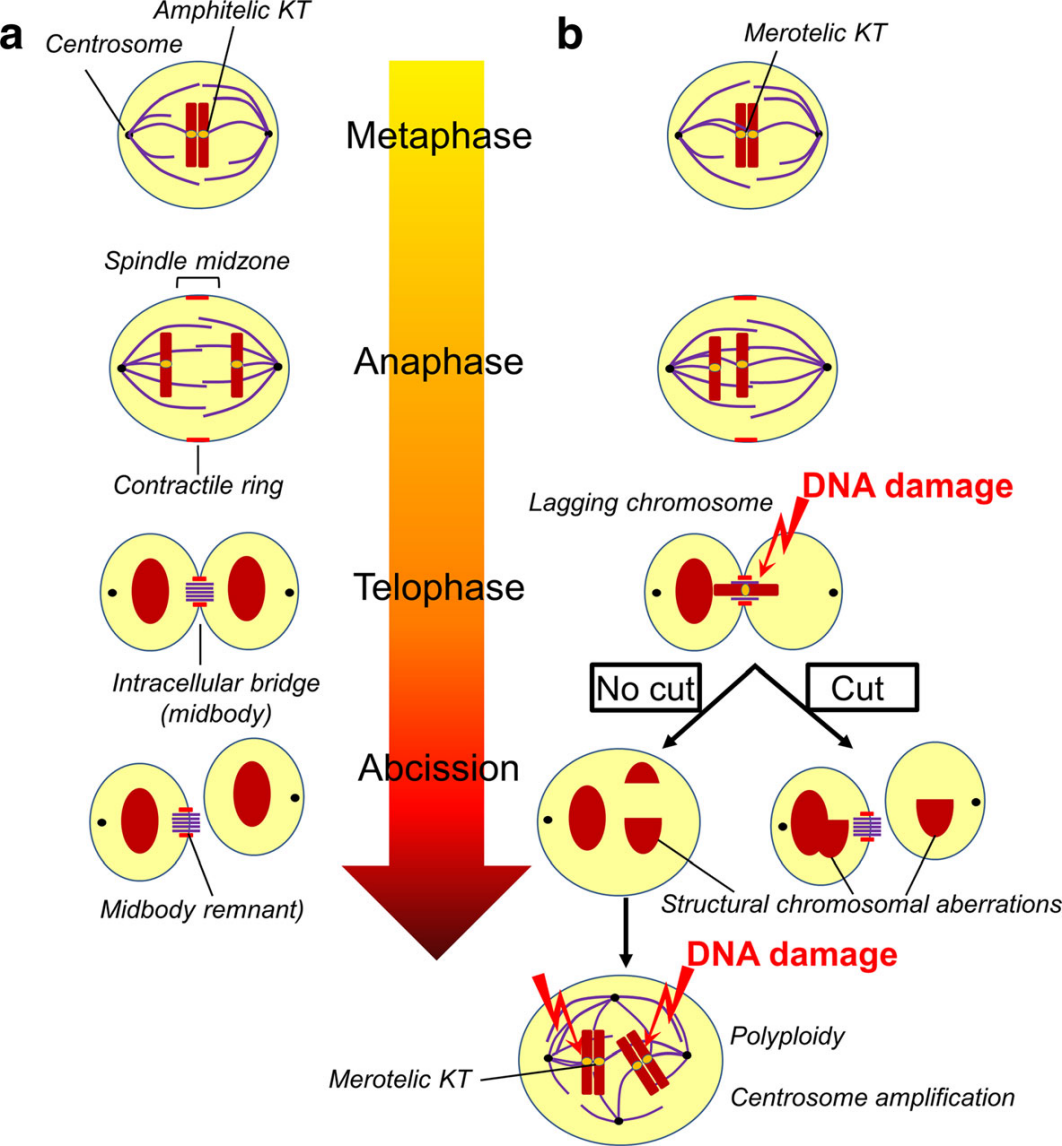
Proposed outline of the Survivin overexpression phenotype. **a**: Normal chromosomal segregation of cells. During metaphase amphitelic kinetochore-microtubule (KT) connections provide the foundation for correct sequential chromosome segregation and abscission of daughter cells. **b**: Overexpression of Survivin stochastically induces false KT connections, such as merotelic connections. The resulting disturbed segregation of chromosomes during anaphase (i.e. lagging chromosomes) can result in trapped chromatin in midbodies. Such affected chromosomes become damaged either by microtubule-generated forces or by cleavage furrow-generated forces. Mechanistically, the “No cut”-pathway of midbody-trapped chromatin could prevent abscission, which promotes the development of polyploid cells with supernumerary centrosomes

Moreover, in this report we show to our best knowledge for the first time, that overexpression of Survivin induces mitotic defects, DNA damage and DDR of glioma cells resulting in increased CIN. By using freshly polyclonal transduced cell instead of using cell clones we excluded proviral position effects and were able to monitor induction of CIN. Mechanistically, overexpression of Survivin stochastically induces false kinetochore-microtubule (KT) connections, such as merotelic KT connections, and trapped chromatin (i.e. lagging chromosomes) at the cleavage furrow during cytokinesis as outlined in Fig. 8. Such affected chromosomes become damaged either by microtubule-generated forces or by cleavage furrow-generated forces [72, 73]. Damaged chromosomes eventually induce a DDR leading to a transient G_1_-arrest and attenuated S-phase entry in p53 wild type cells. In addition, the “No cut”-pathway of midbody-trapped chromatin could prevent abscission of daughter cells and might promote the development of polyploid cells [74]. In line with this notion, the observed DNA damage was only moderate in Survivin-transduced glioma cells as determined by analysis of γH2AX and pDNA-PKcs foci. However, cells were able to repair DNA damage by NHEJ leading to novel structural chromosomal aberrations. Our results may have general implications when investigating Survivin’s cellular functions by the use of ectopically overexpressed Survivin or mutants, since karyotype-specific effects, especially when using cell clones, cannot be fully excluded.

Intriguingly, the Survivin-induced CIN was not sufficient to increase the tumorigenicity of U251-MG glioma cells subcutaneously transplanted in immunodeficient NMRI-Foxn1^nu^/Foxn1^nu^ mice. This might be in part due to inefficient angiogenesis in U251-MG xenografts [58, 59]. To overcome this limitation and to furthermore rule out the possibility that the transduction procedure or residual NK cell activity of NMRI-Foxn1^nu^/Foxn1^nu^ mice contributed to the insufficient tumor growth of mock-transduced U251-MG and U251-MG^Survivin^ cells, we additionally overexpressed mycN, which besides an increased oncogenic signaling induces a metabolic switch to pseudohypoxic glycolysis [60, 68]. As expected from the aforementioned studies, ectopic expression of mycN and simultaneous ectopic expression of mycN and Survivin efficiently produced hyperproliferative gliomas. The ectopic expression of Survivin resulted again in increased CIN in U251-MG^mycN/Survivin^ cells but also in decreased apoptosis when compared to U251-MG^mycN/C^ cells eventually resulting in shortened tumor latency. Furthermore, ex vivo cultivation of tumor-derived U251-MG^mycN/Survivin^ cells showed significant better spheroid growth and growth in soft agar than tumor-derived U251-MG^mycN/C^ cells and when re-transplanted into immundeficient mice produced tumors with significantly shortened latency. Noteworthy, it has been shown that ectopic overexpression of Survivin promotes tumor growth and metastasis via Akt/PKB- and NFκB-dependent upregulation in genes involved in invasion and by FAK- and Src-dependent anchorage-independent growth [75, 76]. This might explain the increased contact-uninhibited cell growth of mycN and Survivin-transduced U251-MG cells and decreased tumor latency when compared to U251-MG cells only transduced with mycN.

## Conclusions

Collectively, our results demonstrate that overexpression of Survivin increases tumorigenicity of mycN-transduced U251-MG glioma cells by combined induction of CIN and prevention of apoptosis. Whether, prevention of apoptosis is directly or indirectly linked to the IAP function of Survivin remains to be elucidated. In this regard, it appears conceivable that ongoing DNA damage and employment of the cellular DNA repair machinery eventually might lay the foundation for radioresistance of glioblastoma. Future analyses of gliomas will show whether increased Survivin-induced DNA damage and CIN impacts radioresistance in gliomas. In summary, the data presented here shed a new light on Survivin in tumor progression and hints at its novel role in adaptive evolution of cancer cells.

## Additional files

Additional file 1: Figure S1. Indirect immunofluorescence analyses demonstrating typical localization of endogenous and ectopic Survivin at kinetochores. Depicted are representative images of SVGp12 cells in metaphase after transduction of control plasmid (first panel) or after transduction with Survivin vector (second panel). Arrowhead depicts an additional metaphase plate in the Survivin-transduced cell. From left to right: DAPI (DNA), FITC (Survivin), WGA-TexasRed (membrane) and merge. (TIFF 1364 kb)

Additional file 2: Figure S2. **a**: Proliferation index of the U251-MG and SVGp12 cells transduced with Survivin and empty vector controls. Depicted are mean values ± SD. **p < 0.01. **b**: BrdU-incorporation in U251-MG and SVGp12 cell fractions with DNA content >4n. Depicted are mean values ± SD. **p < 0.01. All data were collected 72 h after transduction of cells. (TIFF 117 kb)

Additional file 3: Figure S3. Western blot analysis of HCT116 and HCT116^p53−/−^ cell lysates after transduction of Survivin and control vectors. Membranes were probed with anti-p53 (53 kDa), anti-p21^waf/cip^ (21 kDa), anti-p53(S15) (53 kDa) and anti-γH2AX (16 kDa) antibodies. Membranes were re-probed with α-actin (42 kDa) to confirm equal loading. After densitometric analysis the relative expression levels of proteins in Survivin-transduced cells (fold increase) were compared to controls. (TIFF 243 kb)

Additional file 4: Figure S4. Indirect immunofluorescence analyses images of Survivin- and mock-transduced cells stained with a monoclonal antibody specific for phosphoDNA-PKcs. Nuclei were counterstained with DAPI. **a**: Representative images of SVGp12 cells. The Survivin-transduced cell contains multiple containing phosphoDNAPKcs foci **b**: Representative images of transduced U251-MG cells. Note the Survivin-transduced multinuclear U251-MG cell containing numerous phosphoDNAPKcs foci in the nuclei. Magnification bars: 10 μm. Data were collected 72 h after transduction of cells. (TIFF 2132 kb)

Additional file 5: Figure S5. SKY-Analysis showing chromosomal instability (increased numerical and structural chromosomal aberrations) in sorted tumor Survivin-overexpressing U251-MG cells compared to sorted mock-control cells. Representative karyograms of mock-control (upper figure) and Survivin-overexpressing cells (lower figure) with white arrows indicating clonal aberrations already present in the parental cell line. Survivin-overexpressing U251-MG show additional non-clonal structural changes indicated by purple arrows. (TIFF 1830 kb)

## Abbreviations

ATM: Ataxia telangiectasia mutated
BIR: Baculoviral inhibitor of apoptosis repeat
CHK2: Checkpoint kinase 2
CIN: Chromosomal instability
CPC: Chromosomal passenger complex
CPP: Chromosomal passenger protein
DDR: DNA damage response
H3: Histone 3
IAP: Inhibitor of apoptosis protein
INCENP: Inner centromere protein
Ipl1: *Saccharomyces cerevisiae* orthologue of Aurora B kinase
MgcRacGAP: GTPase-activating protein for Rac and Cdc42
NHEJ: Non-homologous end joining
PI3K: Phosphatidylinositol-3-kinase
PTEN: Phosphatase and tensin homolog
RING: Really interesting gene
RNAi: RNA interference
SHCBP1: SHC SH2-domain binding protein 1
SKY: Spectral karyotyping
Sli15: *Saccharomyces cerevisiae* orthologue of INCENP
SV40: Simian virus 40
