# Supplementary figures and images for: Chromosomal instability induced by increased BIRC5/Survivin levels affects tumorigenicity of glioma cells
Source: BMC Cancer. 2017 Dec 28;17:889. doi: 10.1186/s12885-017-3932-y (PMC5745881; doi:10.1186/s12885-017-3932-y)

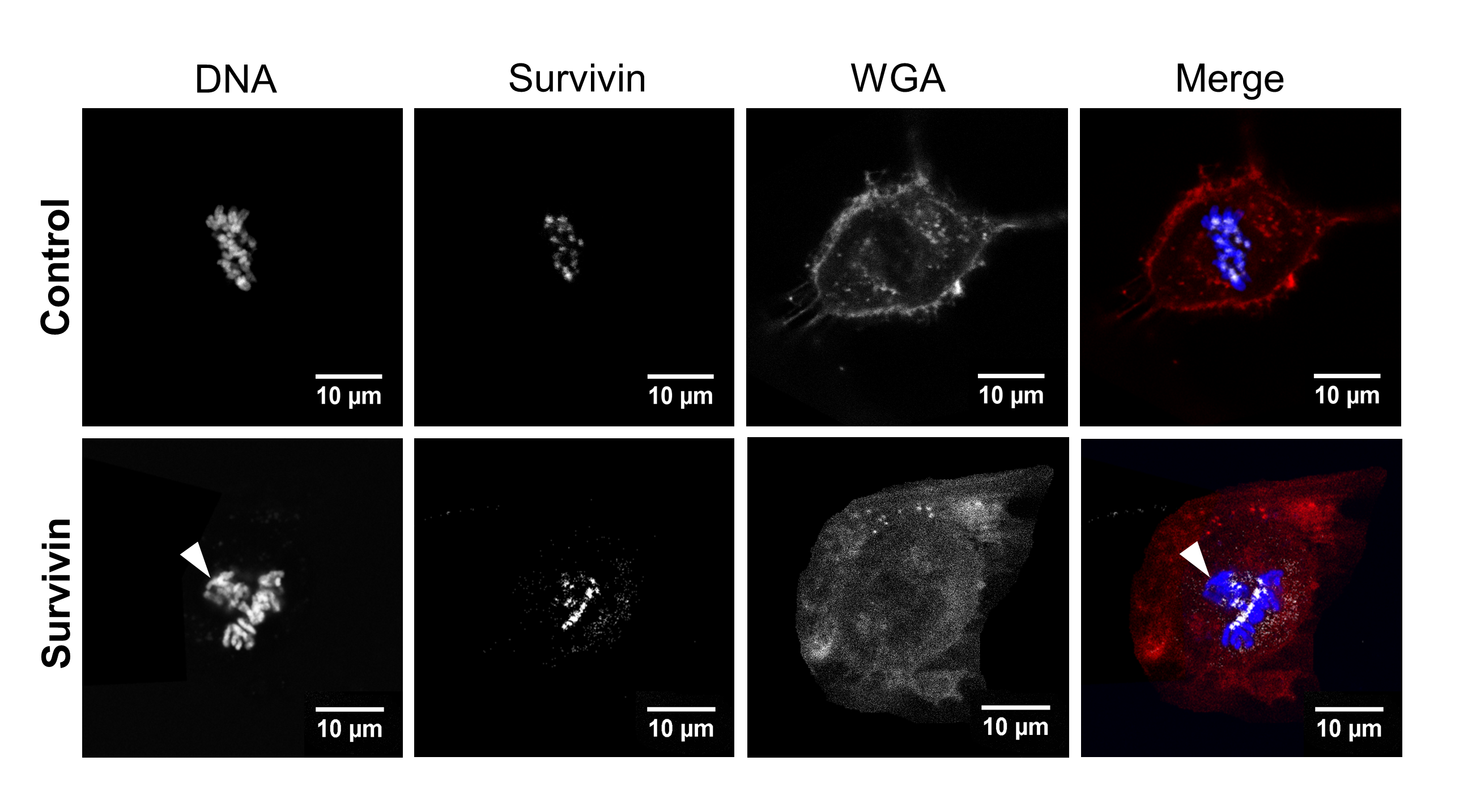

Supplement: Supplementary file 1 — Indirect immunofluorescence analyses demonstrating typical localization of endogenous and ectopic Survivin at kinetochores. Depicted are representative images of SVGp12 cells in metaphase after transduction of control plasmid (first panel) or after transduction with Survivin vector (second panel). Arrowhead depicts an additional metaphase plate in the Survivin-transduced cell. From left to right: DAPI (DNA), FITC (Survivin), WGA-TexasRed (membrane) and merge. (TIFF 1364 kb) [file 12885_2017_3932_MOESM1_ESM.tif]

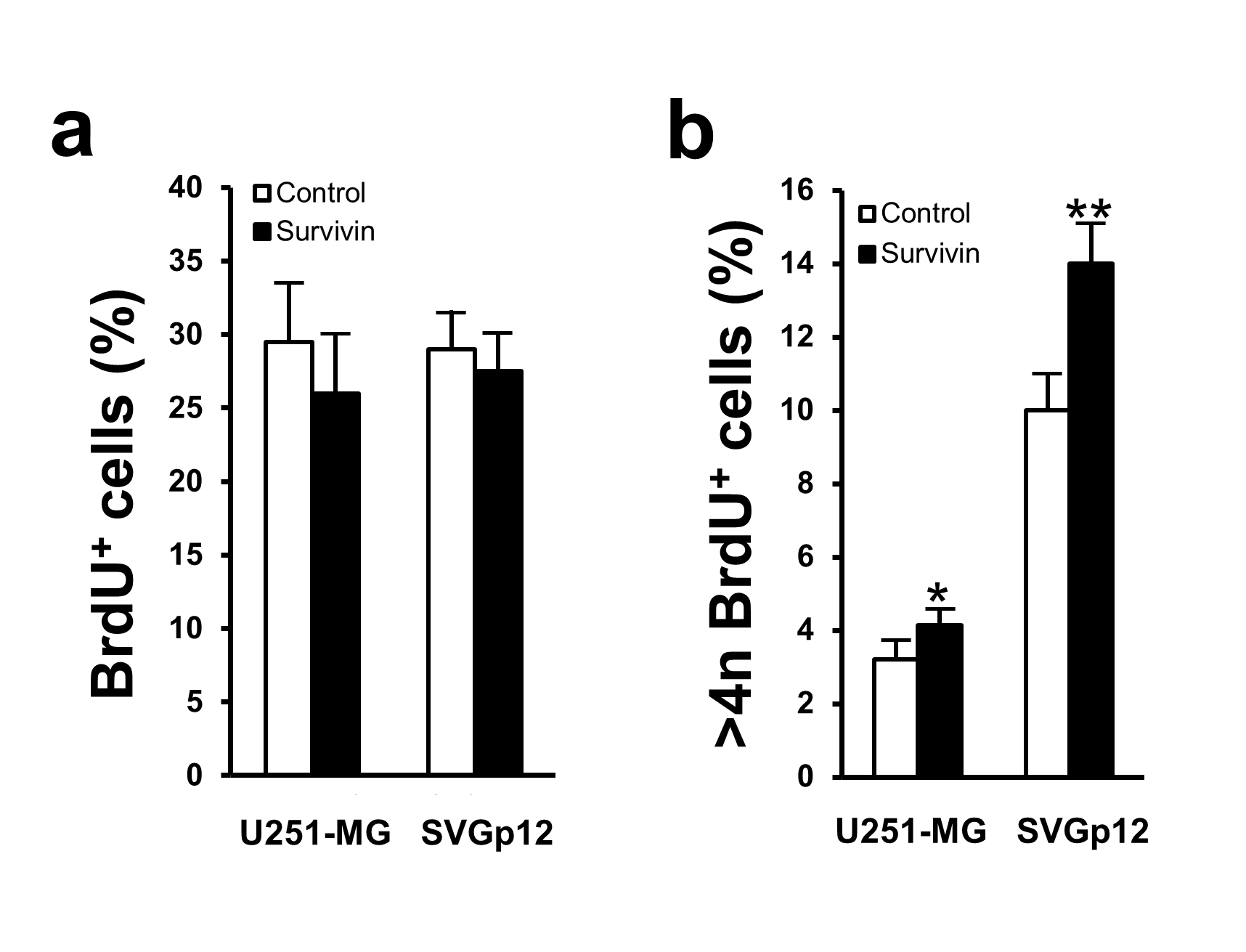

Supplement: Supplementary file 2 — a: Proliferation index of the U251-MG and SVGp12 cells transduced with Survivin and empty vector controls. Depicted are mean values ± SD. **p < 0.01. b: BrdU-incorporation in U251-MG and SVGp12 cell fractions with DNA content >4n. Depicted are mean values ± SD. **p < 0.01. All data were collected 72 h after transduction of cells. (TIFF 117 kb) [file 12885_2017_3932_MOESM2_ESM.tif]

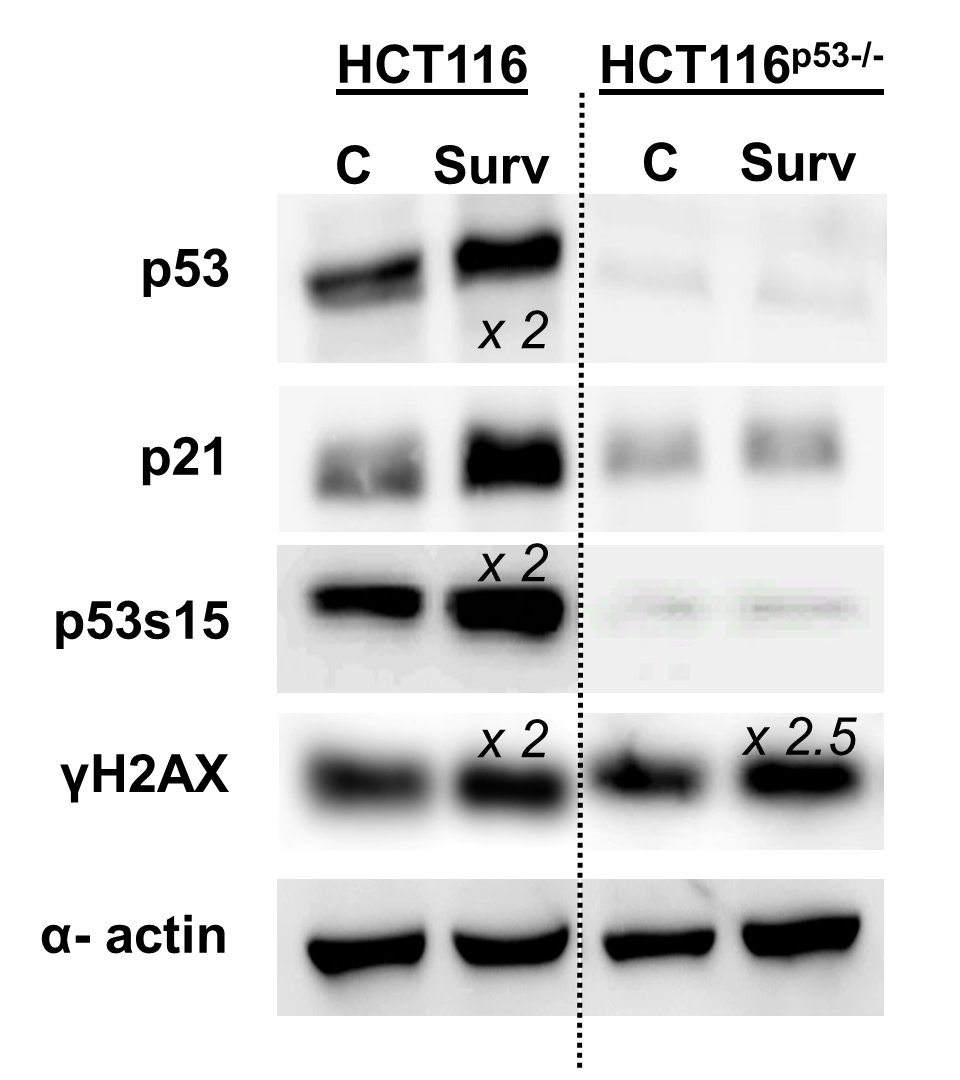

Supplement: Supplementary file 3 — Western blot analysis of HCT116 and HCT116p53−/− cell lysates after transduction of Survivin and control vectors. Membranes were probed with anti-p53 (53 kDa), anti-p21waf/cip (21 kDa), anti-p53(S15) (53 kDa) and anti-γH2AX (16 kDa) antibodies. Membranes were re-probed with α-actin (42 kDa) to confirm equal loading. After densitometric analysis the relative expression levels of proteins in Survivin-transduced cells (fold increase) were compared to controls. (TIFF 243 kb) [file 12885_2017_3932_MOESM3_ESM.tif]

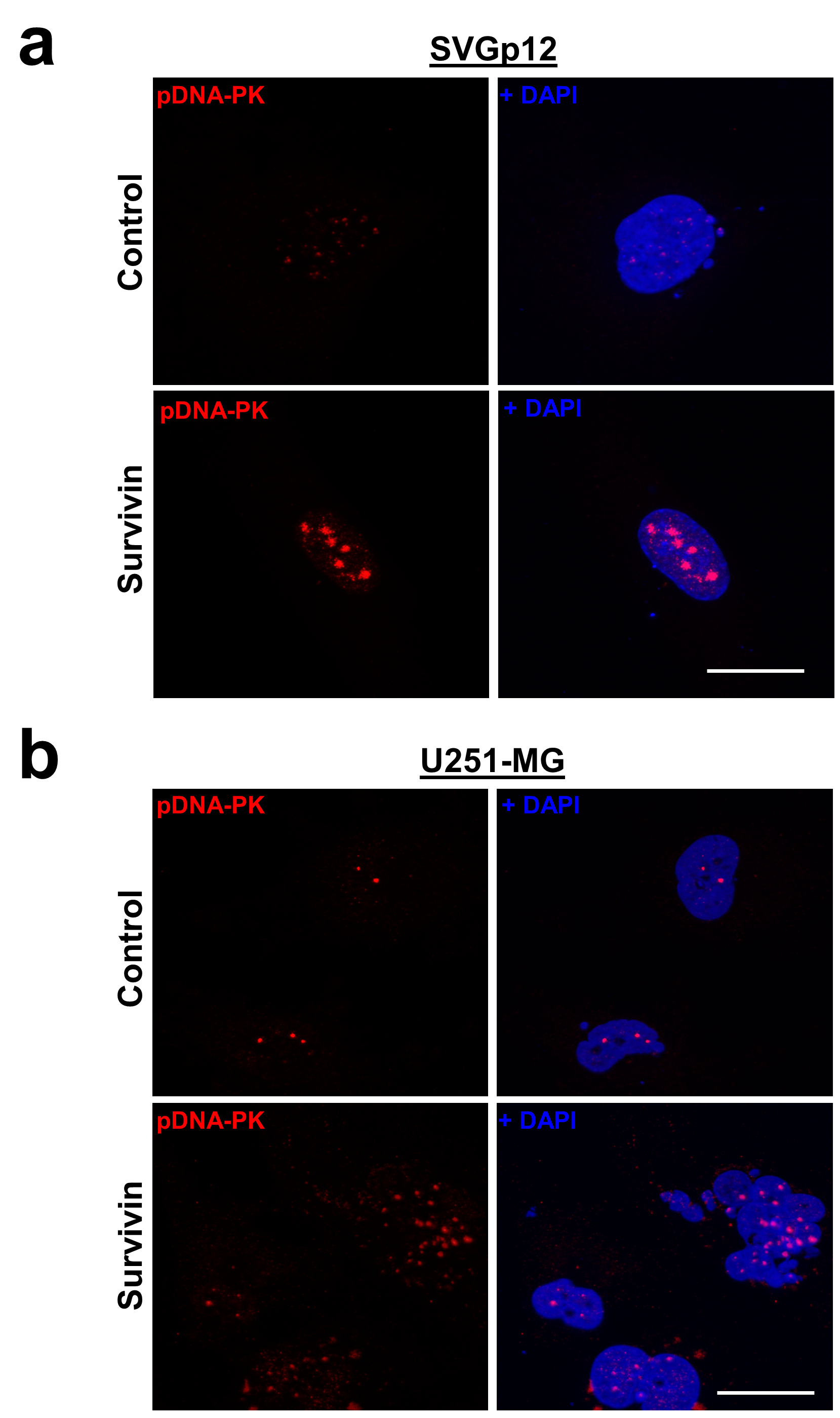

Supplement: Supplementary file 4 — Indirect immunofluorescence analyses images of Survivin- and mock-transduced cells stained with a monoclonal antibody specific for phosphoDNA-PKcs. Nuclei were counterstained with DAPI. a: Representative images of SVGp12 cells. The Survivin-transduced cell contains multiple containing phosphoDNAPKcs foci b: Representative images of transduced U251-MG cells. Note the Survivin-transduced multinuclear U251-MG cell containing numerous phosphoDNAPKcs foci in the nuclei. Magnification bars: 10 μm. Data were collected 72 h after transduction of cells. (TIFF 2132 kb) [file 12885_2017_3932_MOESM4_ESM.tif]

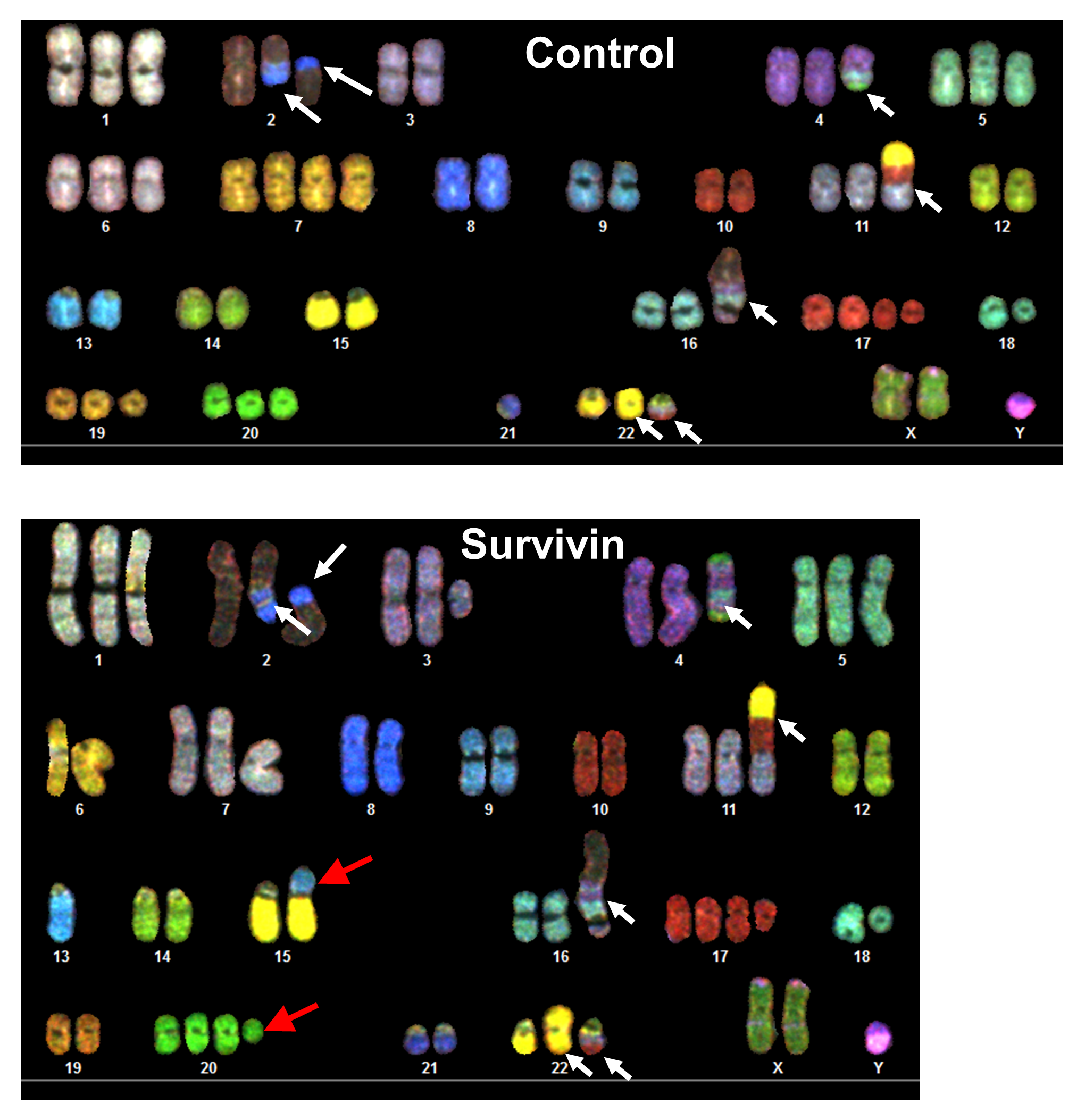

Supplement: Supplementary file 5 — SKY-Analysis showing chromosomal instability (increased numerical and structural chromosomal aberrations) in sorted tumor Survivin-overexpressing U251-MG cells compared to sorted mock-control cells. Representative karyograms of mock-control (upper figure) and Survivin-overexpressing cells (lower figure) with white arrows indicating clonal aberrations already present in the parental cell line. Survivin-overexpressing U251-MG show additional non-clonal structural changes indicated by purple arrows. (TIFF 1830 kb) [file 12885_2017_3932_MOESM5_ESM.tif]
